# Ultrasound radiomics nomogram for predicting large-number cervical lymph node metastasis in papillary thyroid carcinoma

**DOI:** 10.3389/fonc.2023.1159114

**Published:** 2023-06-08

**Authors:** Meiwu Zhang, Yan Zhang, Huilin Wei, Liu Yang, Rui Liu, Baisong Zhang, Shuyi Lyu

**Affiliations:** Department of Interventional Therapy, Ningbo No.2 Hospital, Ningbo, Zhejiang, China

**Keywords:** papillary thyroid carcinoma, lymph node metastasis, radiomics, nomogram, ultrasound

## Abstract

**Purpose:**

To evaluate the value of preoperative ultrasound (US) radiomics nomogram of primary papillary thyroid carcinoma (PTC) for predicting large-number cervical lymph node metastasis (CLNM).

**Materials and methods:**

A retrospective study was conducted to collect the clinical and ultrasonic data of primary PTC. 645 patients were randomly divided into training and testing datasets according to the proportion of 7:3. Minimum redundancy-maximum relevance (mRMR) and least absolution shrinkage and selection operator (LASSO) were used to select features and establish radiomics signature. Multivariate logistic regression was used to establish a US radiomics nomogram containing radiomics signature and selected clinical characteristics. The efficiency of the nomogram was evaluated by the receiver operating characteristic (ROC) curve and calibration curve, and the clinical application value was assessed by decision curve analysis (DCA). Testing dataset was used to validate the model.

**Results:**

TG level, tumor size, aspect ratio, and radiomics signature were significantly correlated with large-number CLNM (all P< 0.05). The ROC curve and calibration curve of the US radiomics nomogram showed good predictive efficiency. In the training dataset, the AUC, accuracy, sensitivity, and specificity were 0.935, 0.897, 0.956, and 0.837, respectively, and in the testing dataset, the AUC, accuracy, sensitivity, and specificity were 0.782, 0.910, 0.533 and 0.943 respectively. DCA showed that the nomogram had some clinical benefits in predicting large-number CLNM.

**Conclusion:**

We have developed an easy-to-use and non-invasive US radiomics nomogram for predicting large-number CLNM with PTC, which combines radiomics signature and clinical risk factors. The nomogram has good predictive efficiency and potential clinical application value.

## Graphical Abstract

US radiomics workflow and study flowchart.

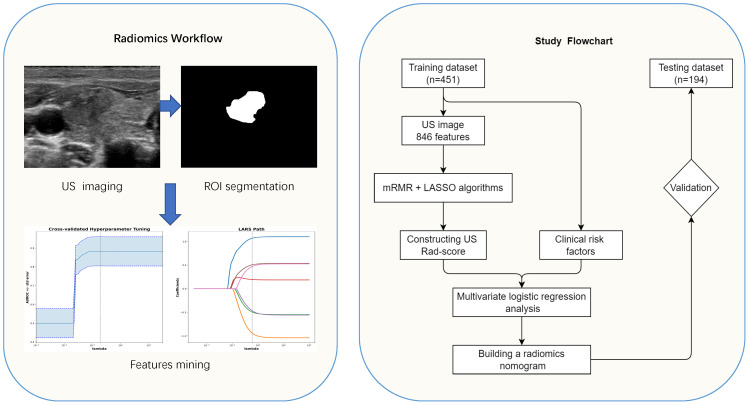

## Introduction

Papillary thyroid carcinoma (PTC) is the most common differentiated thyroid carcinoma (DTC), accounting for 85-90% of thyroid cancer (1). As a result of early detection, the prevalence of PTC has increased significantly, and about 30% of 80% of PTC patients have cervical lymph node metastasis (CLNM) (2). If there is CLNM, the risk of recurrence, distant metastasis, and death will significantly increase. The risk of recurrence in patients with CLNM is 3.33 times higher than in patients without lymph node metastasis (LNM) (3). It has been reported that in the case of small-number CLNM (≤ 5 involved lymph nodes), the recurrence rate is about 5%, while when there are large-number CLNM (> 5 involved lymph nodes), the recurrence rate is about 20% (4). Recurrence is psychologically stressful for patients, and re-operation for thyroid cancer is difficult and the risk of surgical complications such as damage to the recurrent laryngeal nerve and parathyroid glands during re-operation is greatly increased (5). The 2015 ATA guidelines (4) and the 2022 NCCN guidelines (6) recommend that PTC patients with large-number CLNM be treated with 131^I^ after total thyroidectomy. Therefore, preoperative screening of PTC patients with large-number CLNM is of great significance for selecting treatment methods and evaluating prognosis.

Ultrasound is the most convenient and effective method for diagnosing thyroid diseases. Still, due to the influence of trachea gas, thyroid gland, and anatomical location, ultrasound has a low sensitivity in diagnosing CLNM, with only 30%~63% (7, 8). Partial occult LNM cannot be found, and there is a high false negative rate. Previous studies suggested that the irregular shape of the primary PTC was an essential index for predicting CLNM (9), and male, tumor size > 1.0cm were independent risk factors for large-number CLNM (10). However, ultrasound is an operator-dependent imaging modality whose results are prone to subjective interpretation.

Radiomics refers to the high-throughput mining of a large number of quantitative imaging features from medical image data with the help of computer software, realizing tumor segmentation, feature extraction, and modeling, and using statistical and machine learning methods to screen the most valuable imaging features to analyze clinical information (11). It is considered that the radiomics features can reflect the heterogeneity that exists objectively but cannot be recognized by the naked eye to a certain extent, provide important phenotypic information, and describe the tumor microenvironment based on imaging science. Thus, it can assist in evaluating the tumor’s biological characteristics, improve the disease’s diagnostic efficiency, consider the curative effect and predict the prognosis, and contribute to individual and accurate treatment. At present, many studies have confirmed that preoperative ultrasound radiomics has the potential to predict LNM in PTC (12, 13). However, there are few studies on preoperative ultrasound radiomics to predict large-number CLNM in PTC. This study hopes to analyze the radiomics nomogram based on ultrasound of PTC and evaluate its value in predicting large-number CLNM, which helps screen out high-risk patients and provides a basis for further treatment.

## Materials and methods

### Patients

The clinical data of PTC patients treated in Ningbo No.2 Hospital (Ningbo, China) from August 2015 to May 2021 were collected retrospectively. This study was retrospective, without the requirement of informed consent, and was approved by the Human Research Ethics Committee of Ningbo No.2 Hospital (PJNBEY-KY-2019-15001).

**Figure 1:**
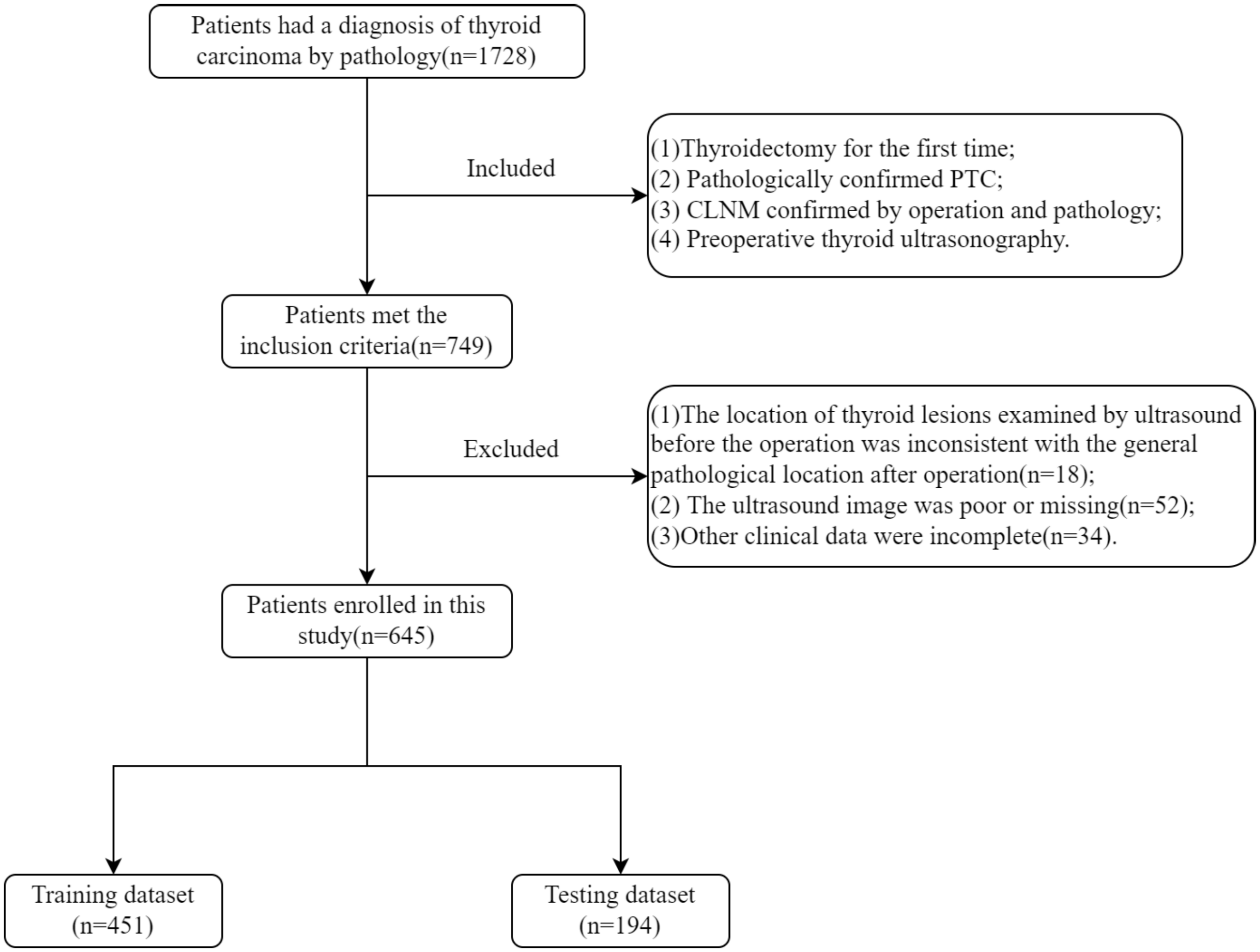
Flow diagram of inclusion criteria and exclusion criteria.

The inclusion criteria were: (1) thyroidectomy for the first time; (2) pathologically confirmed PTC; (3) CLNM confirmed by operation and pathology; (4) preoperative thyroid ultrasonography; (5) patients with multiple cancer lesions, with the largest lesion being the study population. The exclusion criteria were: (1) the location of thyroid lesions examined by ultrasound before the operation was inconsistent with the general pathological location after operation; (2) the ultrasound image was poor or missing, or other clinical data were incomplete, as shown in **Figure 1**.

### Clinical information and ultrasound characteristics

Baseline clinical characteristics, including sex, age, body mass index (BMI, kg/m2), thyroid stimulating hormone (TSH), and thyroglobulin (TG), were collected from electronic medical records.

Before the operation, the MyLab90 color Doppler ultrasound diagnostic system (Esaote S.p.A., Genova, Italy) was used with a LA523 probe(4~13MHz). Select the thyroid examination conditions preset by the machine, observe the target lesions in multiple sections, adjust the gain, depth, dynamic range, and focus area at any time in the examination process to obtain the best imaging effect, and then store the image in the workstation in DICOM format. Ultrasound examination was carried out by a radiologist who had worked for more than 5 years. Two radiologists with more than 8 years of ultrasound experience interpreted all ultrasound image features. They recorded the characteristics of ultrasound images. Including the size of the thyroid nodule (maximum diameter of the tumor), the location of the nodule [including the superior pole of the thyroid (upper 1/3 of the gland), the middle of the thyroid (middle 1/3 of the gland), the inferior pole of the thyroid (inferior 1/3 of the gland), isthmus], Hashimoto Thyroiditis (HT), multifocality, shape, margin, echogenicity, homogeneity of the internal echo, aspect ratio (≥1 vs. <1), calcifications [macrocalcification (>1 mm), microcalcification (≤1 mm), and absent], capsule invasion(capsular abutment by the nodule and loss of the echogenic capsule) and other ultrasonic observations.

### Surgical technique

All patients diagnosed with thyroid cancer undergo routine bilateral central neck dissection, regardless of disease stage or tumor size. Additional lateral lymph node dissection was required for patients with clinically suspected lateral LNM confirmed by preoperative fine needle aspiration (FNA) or intraoperative frozen biopsy.

### Region of interest segmentation and feature extraction

The ultrasound DICOM image data were imported into ITK-SNAP (version 3.8, http://www.ITKSNAP.org), and the region of interest (ROI) was manually delineated on the largest level of grayscale ultrasound images of primary PTC. All segmentation was performed by two radiologists and one radiologist (twice) without knowing the pathological results of cervical lymph nodes. They have more than 8 years of experience in thyroid nodule ultrasonography. The image was preprocessed before feature extraction, including gray standardization, discretization processing, and image resampling. Then the pyradiomics software package (v3.0) was used to automatically extract the radiomics features that reflect phenotypic features, including texture, morphology, intensity-based, Laplace-Gaussian, and wavelet radiomics features. Radiomics features followed the Image Biomarker Standardization Initiative (IBSI, https://ibsi.readthedocs.io/en/latest/). The interclass correlation coefficient (ICC) was used to evaluate the inter-observer and intra-observer consistency of feature extraction. Only the radiomics features with ICC > 0.8 can enter the next step of feature selection and analysis.

### Feature selection and radiomics signature construction

The patients were randomly assigned to the training and testing datasets according to the proportion of 7:3. Spearman’s correlation coefficient was used to calculate the correlation and redundancy between features. The redundancy feature when Spearman’s correlation coefficient≥0.8 was eliminated. Then minimum Redundancy-Maximum Relevance (mRMR) and Least Absolution Shrinkage and Selection Operator (LASSO) regression were used to screen the features in order to reduce the candidate radiomics features that may be associated with large-number CLNM. The radiomics signature score formulas were generated by combining the linear combination of the selected features, and these features were weighted by their respective LASSO algorithm. The radiomics signature score of each patient in the training dataset was calculated by using the formula.

### The establishment of the US radiomics nomogram

In the training dataset, univariate analysis, Student’s T-test (continuous variables), or Chi-squared test (categorical variables) were used to determine the clinicopathological factors related to large-number CLNM. The selected risk factors were further analyzed by multivariate logistic regression analysis, with a liberal p-value less than 0.05 as the retention standard, and the backward step-by-step selection method was used to select the final independent predictors of large-number CLNM. Then, a radiomics nomogram was established by combining radiomics signature and clinical risk factors.

### Performance of the US radiomics nomogram

In the training and testing dataset, the receiver operating characteristic (ROC) curve of the US radiomics nomogram was plotted. The discriminative performance of the radiomics nomogram was evaluated using the area under the curve (AUC). The calibration curve was used to evaluate the effectiveness of the radiomics nomogram. Decision curve analysis (DCA) was used to evaluate the clinical value of the radiomics nomogram.

### Statistical analysis

SPSS 26.0 ((IBM Corp., Armonk, NY, USA) was used for statistical analysis. The continuous data with normal distribution were expressed as mean ± standard deviation (SD) and analyzed using independent samples t-test; otherwise, Mann-Whitney U test was used. The categorical data were expressed as n (%) and analyzed using the chi-square test or Fisher’s exact test. In multivariate logistic analysis, large-number CLNM were used as dependent variables, and the factors with statistically significant differences in univariate analysis were taken as independent variables. Machine learning methods and radiomics were implemented using the Scikit-Learn package of Python 3.8.1. The nomogram and calibration curves of the diagnosis model were drawn by the matplotlib package of Python 3.8.1. DCA was used to evaluate the clinical usefulness of the model by quantifying the net benefit under different threshold probabilities. P < 0.05 was considered to be statistically significant.

## Results

### Clinical characteristics

A total of 645 patients were enrolled in this study, including 102 patients with large-number CLNM and 543 patients with small-number CLNM. The positive rates of large-number CLNM in the training dataset and testing dataset were 14.9% (67/451) and 18.0% (35/194), respectively, and there was no significant difference between the two datasets (P=0.309). There was no significant difference in other clinical characteristics between the two datasets (all P > 0.05), as shown in **Table 1**.

**Table 1:** Baseline clinical and US imaging characteristics of patients with PTC.

Characteristics	Total (*n*=645)	Training dataset(*n*=451)	Testing dataset(*n*=194)	*P value*
Age(Y, x ± s)	43.96 ± 12.50	43.639 ± 12.30	44.691 ± 12.91	0.314
BMI(kg/m^2^, x ± s)	23.18 ± 3.34	23.191 ± 3.43	23.146 ± 3.13	0.916
TG(ng/ml, x ± s)	15.88 ± 32.57	14.827 ± 31.28	18.314 ± 35.27	0.270
TSH(mU/L, x ± s)	1.98 ± 2.62	2.026 ± 3.03	1.870 ± 1.20	0.866
Tumor size(mm, x ± s)	7.11 ± 4.99	7.150 ± 5.28	7.018 ± 4.25	0.446
Gender [n (%)]				0.437
Female	508 (78.8)	351 (77.8)	157 (80.9)	
Male	137 (21.2)	100 (22.2)	37 (19.1)	
HT [n (%)]				0.383
No	503 (78.0)	347 (76.9)	156 (80.4)	
Yes	142 (22.0)	104 (23.1)	38 (19.6)	
Multifocality [n (%)]				0.350
No	471 (73.0)	324 (71.8)	147 (75.8)	
Yes	174 (27.0)	127 (28.2)	47 (24.2)	
Location [n (%)]				0.537
Upper	164 (25.4)	118 (26.2)	46 (23.7)	
Middle	209 (32.4)	140 (31.0)	69 (35.6)	
Lower	201 (31.2)	149 (33.0)	52 (26.8)	
Isthmus	71 (11.0)	44 (9.8)	27 (13.9)	
Shape [n (%)]				0.746
Regular	207 (32.1)	147 (32.6)	60 (30.9)	
Irregular	438 (67.9)	304 (67.4)	134 (69.1)	
Margin [n (%)]				0.651
Smooth	293 (45.4)	208 (46.1)	85 (43.8)	
Irregular	352 (54.6)	243 (53.9)	109 (56.2)	
Echogenicity [n (%)]				0.735
Isoechoic or Hyperechoic	38 (5.9)	28 (6.2)	10 (5.2)	
Hypoechoic	607 (94.1)	423 (93.8)	184 (94.8)	
Homogeneity [n (%)]				0.541
Homogeneous	435 (67.4)	308 (68.3)	127 (65.5)	
Heterogeneous	210 (32.6)	143 (31.7)	67 (34.5)	
Aspect Ratio [n (%)]				0.698
<1	335 (51.9)	237 (52.5)	98 (50.5)	
≥1	310 (48.1)	214 (47.5)	96 (49.5)	
Calcification [n (%)]				0.727
No	354 (54.9)	245 (54.3)	109 (56.2)	
Yes	291 (45.1)	206 (45.7)	85 (43.8)	
Microcalcification [n (%)]				0.961
Absence	423 (65.6)	295 (65.4)	128 (66.0)	
Presence	222 (34.4)	156 (34.6)	66 (34.0)	
Capsule invasion [n (%)]				0.180
No	502 (77.8)	358 (79.4)	144 (74.2)	
Yes	143 (22.2)	93 (20.6)	50 (25.8)	
Large‐Volume CLNM [n (%)]				0.309
Negative	543 (84.2)	384 (85.1)	159 (82.0)	
Positive	102(15.8)	67 (14.9)	35 (18.0)	

US, Ultrasound; PTC, papillary thyroid carcinoma; Y, year; BMI, body mass index; CLNM, cervical lymph node metastasis; HT, Hashimoto’s thyroiditis; TG, Thyroglobulin; TSH, Thyroid stimulating hormone.

### Feature selection and radiomics signature construction

A total of 846 features were extracted from original gray-scale US images by using the pyradiomics software package. The ICCs of intra-observer and inter-observer reproducibility were good, with 0.814-0.903 and 0.739-0.829, respectively. After mRMR and LASSO analysis, 7 radiomics features were screened out. Then, the radiomics signature was generated by combining the selected features in a linear manner, and the final radiomics features and their weighting coefficients are shown in **Table 2**.

**Table 2:** US radiomics feature and weighted coefficient after mRMR +LASSO regression analysis.

Radiomics Features	Coefficients
log-sigma-0-1-mm-3D_glcm_Idmn	0.476
wavelet2-LH_glcm_JointEntropy	0.227
log-sigma-0-2-mm-3D_firstorder_Kurtosis	0.192
log-sigma-0-1-mm-3D_glcm_ClusterShade	0.174
wavelet-HL_glszm_GrayLevelVariance	-0.212
original_shape2D_Elongation	0.001
wavelet-HH_firstorder_Skewness	0.117

US, Ultrasound; mRMR, minimum Redundancy-Maximum Relevance, LASSO, least absolute shrinkage and selection operator.

### The establishment of the US radiomics nomogram

In the univariate analysis, large-number CLNM was significantly correlated with age, TG level, tumor size, multifocality, homogeneity, aspect ratio, calcification, and microcalcification (all P<0.05). The multivariate logistic regression analysis showed that TG level (Beta:0.018, 95%CI:0.008~0.028, P=0.001), tumor size (Beta:0.175, 95%CI:0.104~0.245, P<0.001) and aspect ratio (Beta: -1.549, 95%CI: -2.783~-0.316, P=0.014) were still independent risk variables for large-number CLNM. The radiomics signature constructed in the training dataset was an independent factor for predicting large-number CLNM before operation (P < 0.001), as shown in **Table 3**.

**Table 3:** Univariate analysis and multivariate analysis of factors associated with large-number CLNM in the training dataset.

Characteristics	Univariate Logistic Regression					Multivariate Logistic Regression				
	Beta	[0.025	0.975]	*P*		Beta	[0.025	0.975]	*P*	
Age	-0.040	-0.071	0.010	0.010	*	-0.029	-0.068	0.007	0.120	
BMI	-0.032	-0.137	0.073	0.547						
TG	0.014	0.006	0.022	0.001	**	0.018	0.008	0.028	0.001	**
TSH	-0.072	-0.354	0.210	0.618						
Tumor size	0.216	0.151	0.281	<0.001	***	0.175	0.104	0.245	<0.001	***
Gender	0.490	-0.261	1.242	0.201						
HT	0.153	-0.640	0.945	0.705						
Multifocality	1.009	0.383	1.634	0.002	**	0.750	-0.016	1.515	0.055	
Location	-0.152	-0.760	0.457	0.625						
Shape	0.050	-0.693	0.793	0.896						
Margin	0.346	-0.358	1.050	0.335						
Echogenicity	-0.812	-1.938	0.315	0.158						
Homogeneity	1.192	0.489	1.895	0.001	**	0.535	-0.258	1.327	0.186	
Aspect Ratio	-1.821	-2.788	-0.855	<0.001	***	-1.549	-2.783	-0.316	0.014	*
Calcification	1.704	0.853	2.556	<0.001	***	0.863	-0.523	2.248	0.222	
Microcalcification	1.590	0.846	2.334	<0.001	***	0.100	-1.185	1.386	0.879	
Capsule invasion	0.552	-0.223	1.327	0.162						
Rad Signature	5.986	5.253	6.719	<0.001	***	4.090	3.206	4.973	<0.001	***
Const.						-3.503	-5.328	-1.678	<0.001	***

PTC, papillary thyroid carcinoma; BMI, body mass index; CLNM, cervical lymph node metastasis; HT, Hashimoto’s thyroiditis; TG, Thyroglobulin; TSH, Thyroid stimulating hormone.

* P<0.05; ** P<0.01; ***P<0.001.

**Figure 2:**
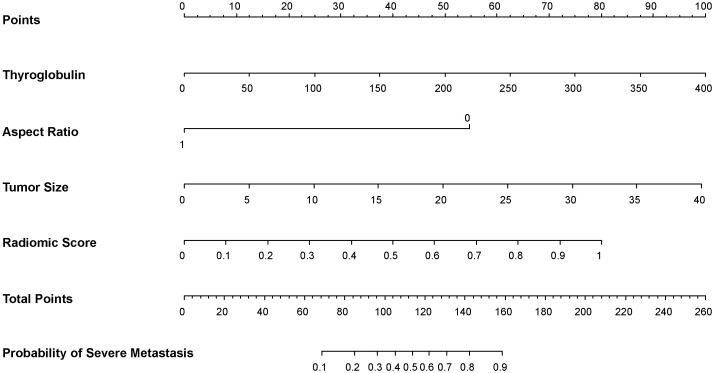
US radiomics nomogram was used to predict the large-number CLNM in PTC patients.

**Figure 3:**
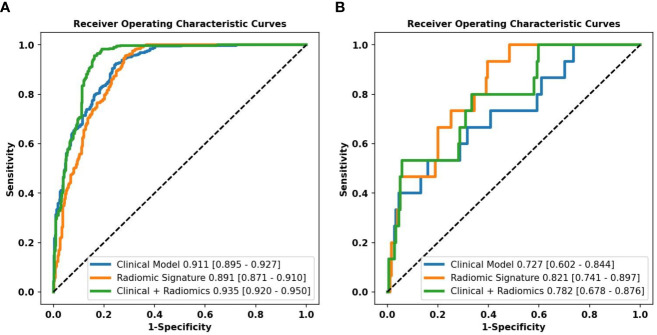
Performance of the different models predicting the large-number CLNM in PTC patients. ROC curves of a clinical model, radiomics signature, and radiomics nomogram for predicting large-number CLNM in the training dataset **(A)** and the testing dataset **(B)**.

**Figure 4:**
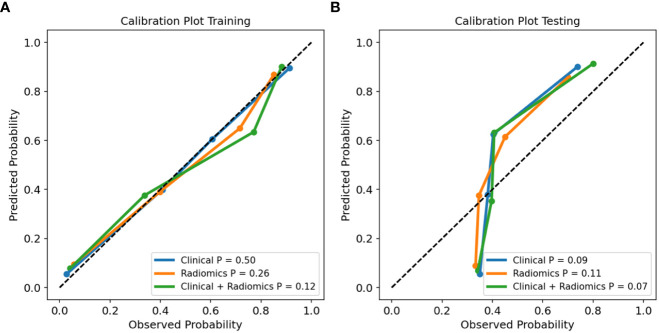
Calibration curves of the clinical model, radiomics signature, and radiomics nomogram in training **(A)** and testing **(B)** dataset.

Combined with clinical risk factors and radiomics signature, a US radiomics nomogram was constructed to predict large-number CLNM in patients with PTC (**Figure 2**). The AUC of the nomogram was 0.935 (95% CI, 0.920-0.950) in the training dataset (**Figure 3A**) and 0.782 (95% CI, 0.678 - 0.876) in the testing dataset (**Figure 3B**). In the training dataset, the calibration curve showed that the prediction curve was in good agreement with the standard curve (**Figure 4A**). In addition, a good consistency of the calibration curve was observed in the testing dataset (**Figure 4B**).

### Clinical significance of US radiomics nomogram

**Figure 5:**
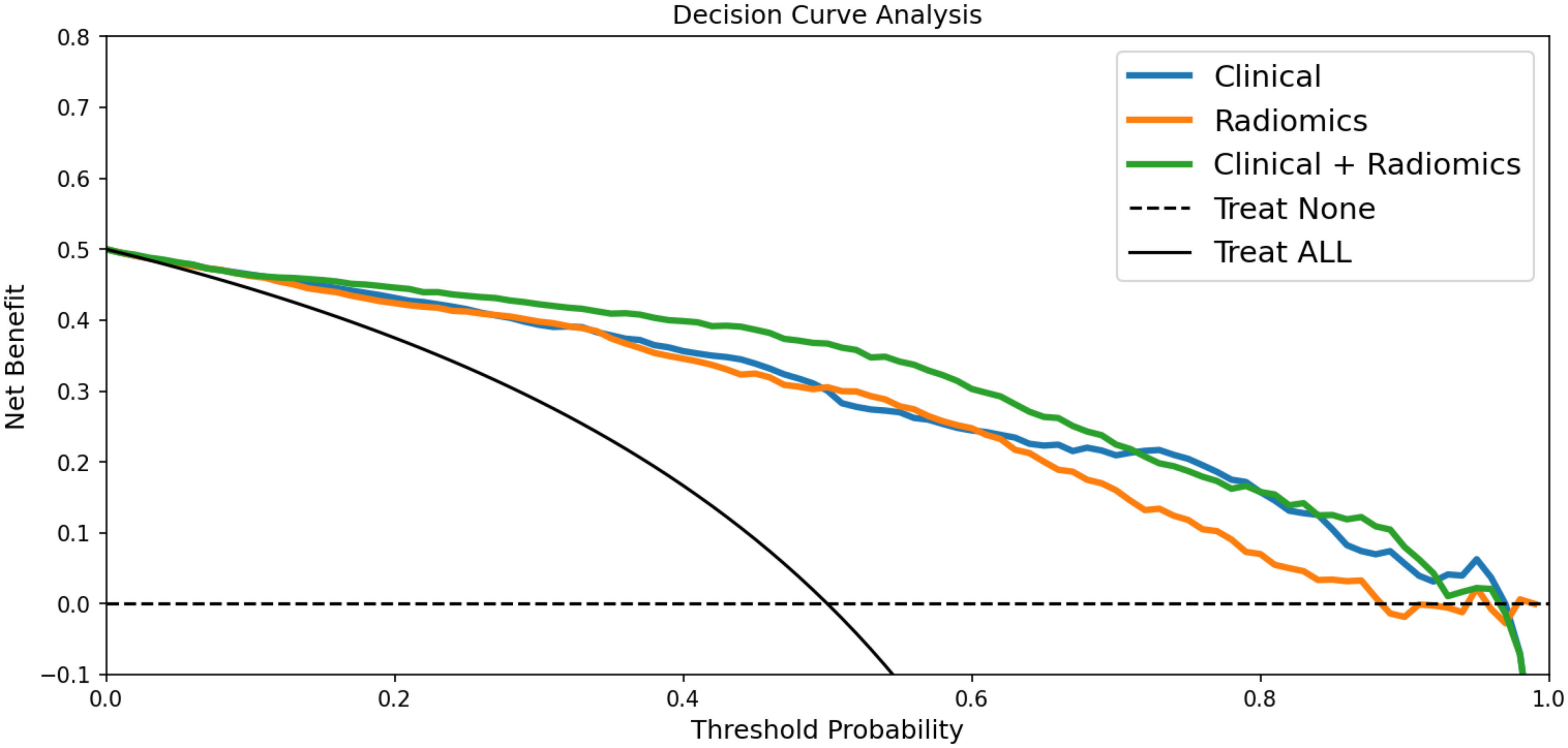
DCA for the clinical model, radiomics signature, and radiomics nomogram in predicting large-number CLNM in PTC patients. According to the threshold probability obtained, the US radiomics nomograms have the most significant net benefit compared with other models or simple strategies such as all-treat and non-treatment.

In the training dataset, the accuracy, sensitivity, and specificity of the US radiomics nomogram were 0.897, 0.956, and 0.837, respectively. In the testing dataset, the model’s accuracy, sensitivity, and specificity were 0.910, 0.533, and 0.943, respectively. Regarding the training dataset’s accuracy, sensitivity, specificity, and AUC, the diagnostic performance of the US radiomics nomogram was better than that of clinical models and radiomics signature. In the testing dataset, the accuracy and specificity of the US radiomics nomogram were better than clinical models and radiomics signature, as shown in **Table 4**. The analysis of DCA found that within the range of reasonable threshold probability, the US radiomics nomogram provided better clinical effectiveness than the clinical model and radiomics signature (**Figure 5**).

**Table 4:** Diagnostic performance of clinical model, radiomics signature, and radiomics nomogram in the training and testing dataset.

Predictive Model	Training Dataset				Testing Dataset			
	Accuracy	Sensitivity	Specificity	AUC	Accuracy	Sensitivity	Specificity	AUC
Clinical model	0.839	0.919	0.759	0.911	0.815	0.533	0.839	0.727
Radiomics Signature	0.835	0.953	0.717	0.891	0.630	0.933	0.603	0.821
Radiomics nomogram	0.897	0.956	0.837	0.935	0.910	0.533	0.943	0.782

AUC, Area under the curve.

## Discussion

LNM is one of the essential clinical factors affecting local recurrence and distant metastasis of tumors. CLNM has a negative effect on overall survival and disease-free survival. In several studies, large-number CLNM was a risk factor for high recurrence and poor prognosis (14). Sugitani et al. reported that patients with more than 5 LNM had a significantly higher risk of recurrence (19% vs. 8%) (15). Similarly, in Leboulleux ‘s study, patients with LNM greater than 10 (21%) or with 6-10 LNM (7%) had a significantly higher risk of recurrence within 10 years than patients with less than 5 LNM (3%) (16). In the 2015 ATA guidelines, more than 5 LNM were one of the criteria for upgrading low-risk PTC to medium-risk PTC, increasing the risk of recurrence of structural diseases by about 15% (4). In addition, in the oncology NCCN clinical practice guide (Thyroid Cancer, 3.2021), large-number CLNM was recommended as one of the adverse pathological features of complete thyroidectomy after lobectomy (6). Therefore, these patients with large-number CLNM should be actively identified and treated because their active treatment significantly affects the prognosis and survival of the disease.

Preoperative ultrasonography of cervical lymph nodes plays an essential role in determining the scope of surgical resection, especially in need for lymph node dissection. Unfortunately, preoperative ultrasound is not sensitive to the diagnosis of LNM and is easily ignored during the examination. Previous studies have explored the relationship between large-number CLNM and ultrasonographic features of PTC. Liu et al. found that sex and tumor size were significantly associated with large-number CLNM (17). Kim et al. found a linear relationship between preoperative TG level, primary tumor size of PTC, and the number of CLNM (r = 0.34, P < 0.001, r = 0.20, P < 0.001) (18). Zhan et al. found that tumor size and “taller than wide” shape were independent risk factors for LNM of thyroid cancer (19). Our results show that large-number CLNM was significantly correlated with age, TG level, tumor size, multifocality, homogeneity, aspect ratio, calcification, and microcalcification in the univariate analysis (all P<0.05). The multivariate logistic regression analysis showed that TG level, tumor size and aspect ratio were still independent risk variables for large-number CLNM. The increase of tumor size will increase the number of lymph node metastasis, and the higher the TG level, the greater the aggressiveness of the tumor,with results similar to those of previous studies (20, 21). Although the ultrasound features mentioned above are encouraging, the ultrasound image features are also based on the subjective judgment of radiologists and lack uniform quantitative standards (22).

Radiomics is a new method of extracting high-throughput quantitative information from standard medical imaging to improve decision-making and has gained interest in cancer research (23, 24). Radiomics is non-invasive, avoids sampling errors in sample biopsies, and allows accurate quantitative analysis of the whole tumor for early diagnosis and prognosis. Radiomics have been explored in the field of thyroid cancer research, such as benign and malignant diagnosis of thyroid nodules (25), prediction of CLNM (26), extrathyroidal invasion of thyroid cancer (27), BRAF-V600E gene mutation (28) and survival prediction (29). Lu et al. developed a nomogram based on enhanced computed tomography (CT) with an AUC value of 0.867, which helps to predict LNM in patients with thyroid cancer (30). However, iodine contrast agents have a risk of allergy in enhanced CT and may delay radioactive iodine, thus limiting its clinical application. Hu et al. established a radiomics model of magnetic resonance imaging (MRI), which has good accuracy in predicting CLNM before operation (31). However, MRI is expensive and takes a long time to examine, so it cannot be routinely used in the clinical treatment of PTC patients.

US radiomics can evaluate the invasiveness of PTC scientifically, objectively, and quantitatively and provide help for clinical choice of treatment. Jiang et al. developed a nomogram that combines the radiomics characteristics of shear wave elastography with clinicopathological parameters, which has a good predictive value for lymph node staging in patients with PTC, AUC was 0.851 and 0.832 in the training dataset and the testing dataset, respectively (32). Park et al. showed that US radiomics of PTC has the potential to predict CLNM (22). However, there are few radiomics models for predicting large-number CLNM in PTC, so there is an urgent need to develop a radiomics model to predict large-number CLNM.

In this study, we tried to develop a radiomics nomogram based on ultrasound images of the primary PTC, which can be used to predict large-number CLNM before operation.

The radiomics signature constructed in the training dataset was an independent factor for predicting large-number CLNM before operation (P < 0.001). On this basis, we combined the radiomics signature and clinical risk factors to construct the US radiomics nomogram. The nomogram can intuitively and individually evaluate the risk of large-number CLNM in patients with PTC. The AUC (0.935, 95%CI: 0.920-0.950) of the nomogram showed higher predictive efficiency than the clinical model in the training dataset, and the AUC (0.782,95%CI:0.678-0.876) of the nomogram was also higher than the clinical model in the testing dataset for judging large-number CLNM. Formulating a lymph node dissection strategy before operation can provide objective evaluation reference for clinicians. The calibration curve shows a high consistency between the predicted and measured values, which shows that the nomogram has good statistical effectiveness.

We applied the DCA method to evaluate whether nomogram-assisted decision-making is beneficial to the prognosis of patients. DCA shows that the net benefit that the predictive model can obtain at different disease risk thresholds is usually used to evaluate the clinical value of the predictive model. Our results show that compared with clinical models and radiomics signatures, the US radiomics nomograms provide better clinical net benefits within the most reasonable threshold probability range.

However, this study still had the following limitations: (1) this study was a retrospective study, and there was an inevitable sample selection bias. (2) the sample size of patients with large-number CLNM was small, and the data between groups may need to be balanced. (3) this study was a single-center study, and there needed to be more external testing and multiple testing of multi-center large sample data to ensure the reliability of the practical application of the radiomics nomogram. Therefore, in the future, we will seek multi-center cooperation to establish a large sample database.

## Conclusion

In a word, we have developed an easy-to-use and non-invasive US radiomics nomogram for predicting large-number CLNM in patients with PTC, which combines radiomics signature and clinical risk factors. The nomogram proposed in this paper has good predictive efficiency and has potential clinical application value.

## Data availability statement

The original contributions presented in the study are included in the article/supplementary material. Further inquiries can be directed to the corresponding author.

## Ethics statement

This study was retrospective, without the requirement of informed consent, and was approved by the Human Research Ethics Committee of Ningbo No.2 Hospital (PJNBEY-KY-2019-15001). Written informed consent from the patients was not required to participate in this study in accordance with the national legislation and the institutional requirements.

## Author contributions

MZ: Data analysis, writing, editing. YZ, RL: Case collection, data collection and analysis. HW, LY: Delineation of the region of interest. BZ: Software, validation. SL: Design, supervision, writing-review and editing of experimental methods. All authors contributed to the article and approved the submitted version.
